# Vacuum-Assisted Microneedle Platforms for Dermal Interstitial Fluid Sampling

**DOI:** 10.3390/pharmaceutics18080926

**Published:** 2026-07-28

**Authors:** Jihyun (Luna) Hwang, Maria T. Dulay, Bruce Schaar, Joseph M. DeSimone

**Affiliations:** 1 Department of Chemical Engineering, Stanford University, Stanford, CA 94305, USA; 2 Department of Radiology, Stanford University, Stanford, CA 94305, USA; 3 Canary Center at the Stanford School of Medicine, Stanford University, Stanford, CA 94305, USA; 4 Stanford Cancer Institute, Stanford University, Stanford, CA 94305, USA

**Keywords:** dermal interstitial fluid (ISF), microneedle array patch, vacuum-assisted ISF sampling, Darcy’s law, point-of-care diagnostics

## Abstract

**Background**: Dermal interstitial fluid (ISF) contains both plasma-derived biomarkers and biomarkers unique to ISF, making it a promising biofluid for painless, scalable, and decentralized liquid biopsy and continuous health monitoring. However, efficient ISF collection remains challenging due to the small accessible fluid volume in the dermis, slow physiological turnover, and stratum corneum. **Methods**: This review reframes dermal ISF sampling as a pressure gradient engineering problem using Darcy’s law. We examine how vacuum-assisted microneedle platforms can effectively drive ISF through the dermal extracellular matrix in a minimally invasive manner. We compare the two architectures: micropore-based and hollow microneedle approaches. **Results**: In the micropore approach, a vacuum chamber is placed over the transient micropores left by withdrawn microneedles, supporting off-device, multi-omic downstream analyses of the collected ISF. The hollow microneedle approach retains the microneedles in the skin and applies vacuum through internal lumens, allowing integration of the vacuum source, microneedles, and biosensors into a single wearable platform for in situ biomarker detection. Comparative studies across these architectures identify the vacuum seal between the device and the skin as the major engineering bottleneck shared by both architectures. **Conclusions**: Vacuum-assisted microneedle platforms provide a practical route for generating pressure gradient-driven ISF transport while preserving minimally invasive skin access. Future development should prioritize device–skin vacuum seal robustness, reproducible ISF recovery across users and skin sites, integrated vacuum sources, scalable fabrication, and usability in clinical or at-home settings.

## 1. Introduction

Interstitial fluid (ISF) is the bodily fluid that bathes cells and tissues. Over the past several decades it has attracted sustained interest as a medium for minimally invasive liquid biopsy that is well suited for continuous, real-time health monitoring [1,2,3]. ISF accounts for roughly three-quarters of the extracellular fluid compartment and 15–25% of total body weight, making it approximately three times as abundant as whole blood by volume [4,5,6]. Formed by transcapillary exchange and cleared by lymphatic vessels, ISF serves as the fluid through which cells communicate via molecular signals, receive nutrients, and dispose of waste products [7,8].

### 1.1. Dermal ISF as a Diagnostic Medium

**Figure 1:**
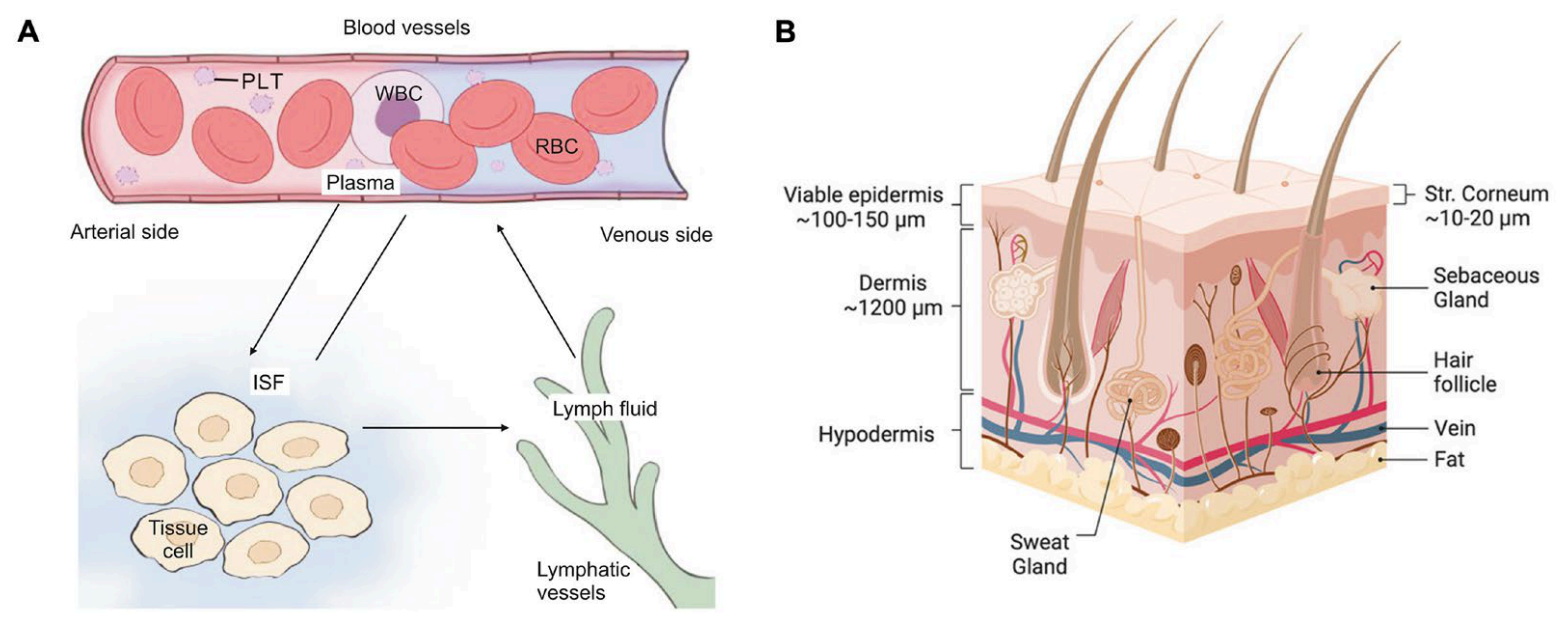
(**A**) Microcirculation between plasma, ISF, and lymph. Reproduced from ref. [14] under a Creative Commons license CC BY-NC-ND 4.0. (**B**) Cross-section layers of the skin. Reproduced from ref. [2] under a Creative Commons license CC BY 4.0.

In the skin, ISF is most abundant in the dermis at approximately 0.4 mL/g of the tissue, which corresponds to roughly 44% of the dermis volume given a typical skin density of 1.1 g/mL [8,9,10]. The dermis hosts two arteriole-fed capillary plexuses whose walls are inherently leaky through multiple extravasation pathways, keeping dermal ISF in continuous exchange with systemic circulation and supplying most of its nutrients, signaling molecules, and biomarkers [8,11]. ISF is then drained by dermal lymphatic capillaries, which prevents the skin from becoming edematous [12]. Under steady-state homeostatic conditions, there is generally net microvascular filtration into the interstitium, followed by uptake into lymphatics. The magnitude and spatial distribution of this flow are governed by hydrostatic and oncotic forces, endothelial permeability, interstitial mechanics and lymphatic function (Figure 1A) [8,13].

The diagnostic value of dermal ISF is supported by its substantial molecular overlap with plasma and serum. Continuous glucose monitoring provides the most established clinical example, which exploits the equilibration of glucose between blood and ISF using minimally invasive electrochemical sensors [15,16,17]. More recent proteomic and metabolomic studies indicate that this overlap extends well beyond glucose, including more than 600 medically relevant protein biomarkers and metabolites such as uric acid, creatinine, vitamins, and fatty acids [6,18,19,20]. These findings suggest that dermal ISF can support diagnostic measurements traditionally performed using blood, while enabling less invasive access. At the same time, ISF is not simply a passive copy of plasma. Several studies have identified a subset of biomarkers unique to or enriched in dermal ISF. Tran et al. identified 113 proteins (3.2% of all identified proteins) that were detected only in ISF in one out of three donors [21]. Samant et al. similarly reported numerous metabolites detected predominantly in ISF and not in blood, including dermatologically derived molecules such as diethanolamine and sphingosine, as well as exogenous compounds absorbed through the stratum corneum (SC), such as trans-cyclohexane-1,2-diol and methyl jasmonate [20]. Dermal ISF therefore offers dual diagnostic value, reflecting systemic physiology through its overlap with plasma while simultaneously including local biomarkers that are often diluted or absent in other biofluids, such as blood, saliva, sweat, tears, and urine.

Beyond its rich biomarker content, dermal ISF has practical advantages as a diagnostic sample. Compared with whole blood, cleanly collected ISF contains few platelets and substantially lower concentrations of coagulation components and therefore generally shows much less visible clot formation, permitting collection without added anticoagulants [22]. Second, when collected correctly without capillary breach, ISF is essentially blood-free, eliminating the hemolysis, cellular debris, and clotting-cascade artifacts that complicate downstream analysis of whole-blood samples [6,19]. Together, these compositional and physical properties establish dermal ISF as a viable medium for minimally invasive liquid biopsy.

Although dermal ISF is diagnostically attractive, its extraction is constrained by the layered anatomy of the skin, the small accessible ISF volume in the dermis, and the slow physiological turnover of dermal extracellular matrix (ECM). To access the dermis, which contains the highest ISF content, an ISF sampling device must traverse the epidermal barrier (Figure 1B) [8]. The epidermis is approximately 100–150 µm thick and consists of four to five layers with various thicknesses and compositions, including the SC, a cornified outer layer that serves as a protective barrier against the external environment [2,8,23]. The SC imposes a transport resistance that is orders of magnitude higher than the underlying viable epidermis and dermis [24]. These skin layers create an initial physical barrier to dermal ISF access. However, even in the thickest regions of the dermis (~3 mm), only ~120 µL of ISF is present per cm^2^ of skin [8]. In addition, the dermal ECM, composed largely of collagen, glycosaminoglycans (GAGs), and proteoglycans, confines bound water and creates high hydraulic resistance to convective fluid flow, further limiting ISF extraction [7,8]. Moreover, unlike blood, which is actively pumped through the circulatory system, dermal ISF is a relatively stationary fluid [8,9]. Friedel et al. noted that the dermal ISF pool is more accurately characterized by lymphatic clearance than by capillary inflow, due to the high hydraulic resistance of the dermal ECM. They estimated that the effective ISF replenishment rate over a 3 mm thick dermis × 1 cm^2^ area is only ~60 nL min^−1^ cm^−2^ based on lymphatic clearance, such that fully replacing the ~120 µL cm^−2^ of accessible dermal ISF would require more than 30 h under idealized conditions [8]. Together, these anatomical and physiological constraints indicate that efficient dermal ISF sampling cannot rely on passive fluid availability alone but, instead, requires an externally imposed driving force capable of bypassing the SC and moving ISF through a resistive and slowly replenished dermal ECM.

### 1.2. Darcy’s Law as a Device Design Framework for Efficient Dermal ISF Sampling

**Figure 2:**
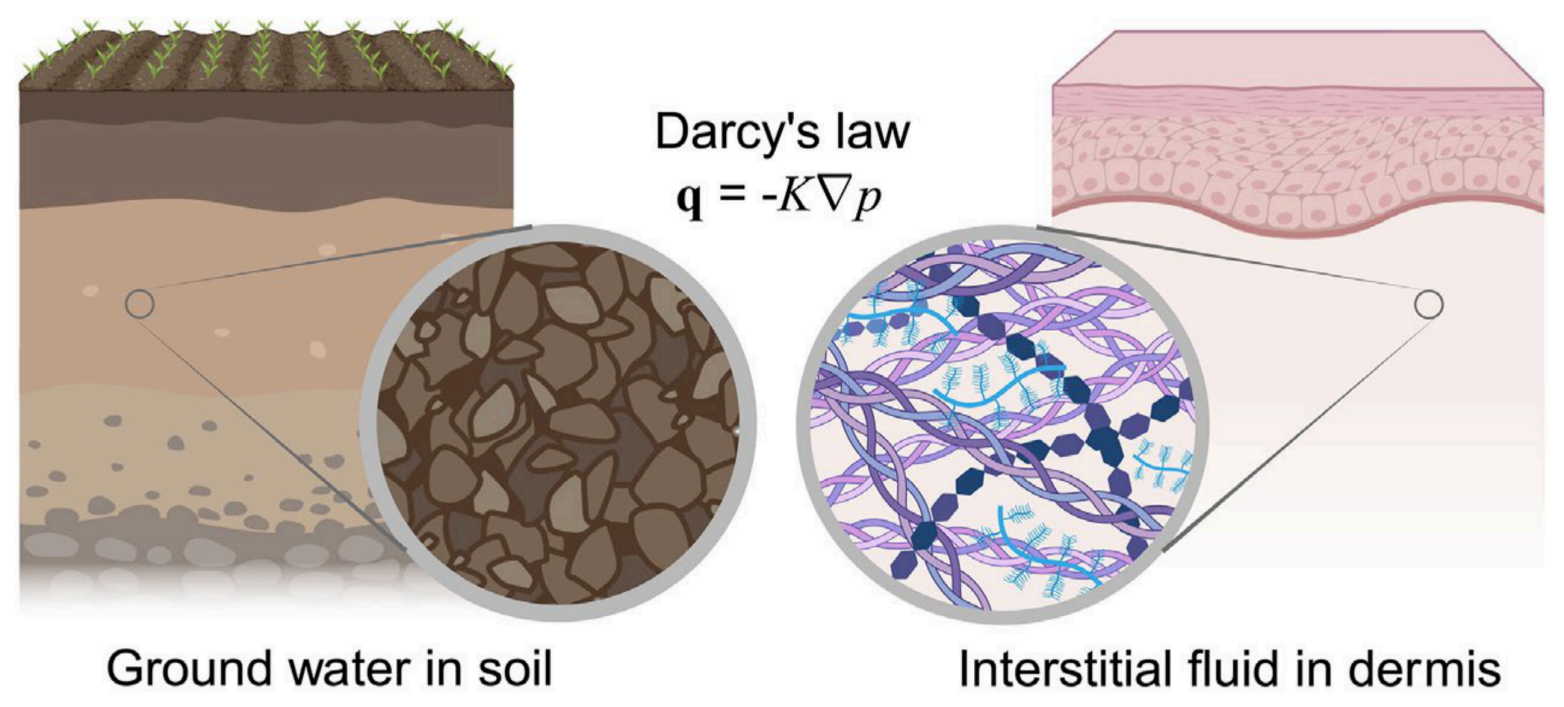
Darcy’s law describing groundwater flow in soil, as well as ISF flow in the dermis. Reproduced from ref. [19] under a Creative Commons license CC BY 4.0.

Because the dermis behaves as a porous matrix with high hydraulic resistance, fluid flow in the dermis can be described using continuum porous-medium theory. Darcy’s law provides this framework by relating ISF flux through the dermal ECM to the pressure gradient applied across it [25,26,27]. Originally derived for groundwater flow through sand beds, Darcy’s law describes a linear relationship between a fluid flow rate through a porous matrix and pressure gradient (Figure 2):

q = −K′∇P = −(K/µ) ∇P(1)

where q is the volumetric fluid flux per unit area, K′ is the hydraulic conductivity, ∇P is a pressure gradient, K is the specific permeability, and μ is viscosity of the fluid phase. In the context of dermal ISF sampling, Darcy’s law identifies two principal determinants of bulk ISF transport (q): the hydraulic conductivity of the dermal interstitium (K′) and the pressure gradient (∇P) imposed by the local physiology or by an ISF sampling device [7,28]. Under this framework, ISF collection can be increased by raising dermal hydraulic conductivity or by increasing the applied pressure gradient.

The first lever, hydraulic conductivity, is governed by ECM composition, organization, and charge [7]. Structurally, the dermis combines a dense fibrillar collagen scaffold with a hyaluronan-rich GAG gel in the interfibrillar space, which together impose steric and electrostatic resistance to water flow [4,7]. Levick (1987) identified collagen as the major contributor of hydraulic resistance in the dermis, while Aukland and Nicolaysen (1981) emphasized the contribution of GAG concentration to resistance against water flow [4,29]. Wiig and Swartz (2012) further described hydraulic conductivity as being governed by the free water volume fraction, where the fraction is set by the balance between swelling forces (e.g., hydrostatic pressure, osmotic pressure, and electrostatic forces from GAGs) and counteracting forces (e.g., cells’ contractile forces and tension in the ECM) [7]. Mena-Lapaix et al. (2025) raised hydraulic conductivity through laser-induced fluid recruitment (LIFR), in which laser pretreatment increased local skin water content before microneedle-assisted suction [30]. In ex vivo porcine skin, dermal pore size increased >5-fold, directly measured hydraulic conductivity increased ~1.5-fold, and ISF extraction was approximately 7-fold higher than in non-hydrated controls. In in vivo human volunteers (forearm), the laser-microneedle-suction system collected up to 55 µL of ISF, an 8-fold improvement over controls without laser pretreatment. The authors attribute the gain to enlarged, water-filled pores in the hydrated dermis, which both lower the resistance to flow and provide expanded pathways available for ISF transport [30]. This study illustrates that increasing dermal hydration can enhance ISF recovery, but such tissue pretreatment may also introduce variability, alter the native sampling environment, and reduce the feasibility of clinical or at-home translation.

The second lever, the applied pressure gradient, is more directly controllable through device design. Hung et al. (2025) targeted the applied pressure gradient as the organizing principle of their device design [19]. Their puncture-out-and-press (POP) microneedle device exploited the spatial distribution of pressure gradient using a perforated collection plate, which creates low pressure at the microneedle-generated puncture sites and high pressure at the surrounding skin, driving ISF from the dermis to the collection plate. In head-to-head comparisons on the same ex vivo human skin at 50 kPa applied pressure, POP collected 1.9–4× more ISF than uniform-pressure controls without the perforated collection plate. Hung et al. further derived a volume–time expression from Darcy’s law, V(t) = V_max_(1 − e^−t/c^), where V_max_ is the maximum volume extraction under the collection conditions and c is a constant, assuming that hydraulic conductivity decreases linearly as ISF is removed from the tissue. This simplified model effectively fits the observed ex vivo collection kinetics, supporting Darcy’s law as a valuable heuristic for designing ISF sampling devices [19].

Despite its utility, Darcy’s law in its classical form rests on several simplifying assumptions that limit its quantitative validity for dermal ISF flow. First, Darcy’s law is a continuum approximation of microscopic fluid flow in a porous medium, in which flow is represented by averaged quantities such as bulk-averaged velocity and hydraulic conductivity of the interstitium. In skin, this approximation is limited because the dermis is structurally heterogeneous at the microscale, and Darcy’s law cannot resolve flow profiles between individual fibers or around cells [28]. While useful for describing average ISF velocity, Darcy’s law is first order with respect to velocity and therefore cannot enforce no-slip boundary conditions at internal solid surfaces such as fiber walls or cell membranes [28]. Second, Darcy’s law is based on the fixed- or rigid-matrix assumption, while the dermis is not a rigid porous bed but a hydrated, deformable ECM that changes in response to applied stress. Consequently, in contrast to the rigid solid assumption underlying Darcy’s law, the ECM porosity and hydraulic conductivity in skin in fact depend on local fluid pressure and solid stress [28]. Third, Darcy’s law describes only the flow of ISF within the dermal interstitium and provides no information on how that fluid is replenished when ISF sampling depletes the local pool [28].

These limitations can be partially addressed by related transport frameworks. First, the Brinkman equation can address the no-slip-boundary limitation of Darcy’s law by adding a second-order viscous term, which can then satisfy no-slip conditions at the surface of solid bodies within the flow field, such as cells embedded in a porous matrix [28,31]. However, the Brinkman equation still considers the porous medium, or the fibers in this context, as a continuum rather than individual fibers [28]. In interstitial physiology, the Brinkman equation is used to estimate the average shear stress over the surfaces of cells embedded in a three-dimensional matrix [32]. Second, the rigid-matrix approximation can be addressed by Biot’s poroelasticity theory, which couples the local fluid pressure to deformation of the solid matrix [33]. Biot’s theory is more appropriate for skin because applied pressure or suction can deform the skin and alter local porosity and permeability during ISF collection [34]. Lastly, the Starling principle addresses the replenishment limitation of Darcy’s law by describing transcapillary fluid exchange. Starling proposed in 1896 that walls of capillaries are semipermeable membranes and that transcapillary fluid movement is governed by the imbalance between capillary hydraulic pressure and colloid osmotic pressure (COP) [35,36]. In 2010, Levick and Michel revised the Starling principle to account for the glycocalyx as the semi-permeable layer of endothelium, replacing the bulk interstitial COP with the COP of the ultrafiltrate on the underside of the glycocalyx [36]. The revised principle supports the view that filtered fluid returns to the circulation primarily through lymphatic drainage rather than venular reabsorption [36,37]. For ISF sampling, this suggests a physiological upper bound on the rate at which transcapillary inflow can replenish a depleted ISF, which aligns with how Friedel et al. calculated the dermal ISF replenishment rate based on lymphatic clearance rate [8]. While the limitations of Darcy’s law in describing dermal ISF flow can be addressed through extensions such as Brinkman’s equation, Biot’s poroelasticity theory, and the revised Starling principle, these frameworks primarily capture intrinsic biophysical properties of the dermis that are not readily controllable from a device design standpoint. Consequently, the most relevant device design parameters remain those explicitly represented within Darcy’s law: the hydraulic conductivity and the applied pressure gradient. In principle, hydraulic conductivity could be modulated through skin pretreatment strategies such as the LIFR [30]; however, such interventions may perturb the native composition of ISF. By contrast, the pressure gradient can be directly imposed through device design without requiring separate skin pretreatment, making it a more practical and controllable parameter for engineering ISF sampling devices. This can be achieved through externally applied forces, including suction and positive pressure. The development of ISF sampling devices can therefore be framed primarily as the challenge of establishing a sufficient pressure gradient across the dermis while minimizing additional tissue perturbation.

In this review, we establish Darcy’s law as a pragmatic framework for the design of dermal ISF sampling devices. Within this framework, we identify vacuum-assisted microneedle platforms as a practical means of generating a sufficient pressure gradient across the dermis while minimizing skin invasion. We group these platforms into two major architectures (micropore-based and hollow microneedle-based systems), and compare their design principles, performance, and translational limitations.

## 2. Strategies to Maximize the Pressure Gradient in the Dermis

To efficiently establish a pressure gradient across the dermis that is large enough to overcome the ECM’s hydraulic resistance and the slow physiological turnover of the interstitial pool, an ISF sampling device must first traverse the SC [7,8,30]. When the SC is intact, an applied pressure drops largely, and direct ISF sampling becomes highly inefficient regardless of the applied magnitude [30].

Direct dermal access can be achieved using invasive methods such as suction blistering and microdialysis. Suction blistering applies sustained negative pressure to separate epidermis from the dermis and can collect relatively large volumes of suction blister fluid, but the procedure is slow and can damage the skin, such as bruising, petechiae, and histological tearing damage [38,39,40]. Similarly, microdialysis can directly access the dermis and continuously sample soluble analytes in the ISF; however, the probe insertion is invasive, requires a stabilization period, and often recovers diluted analyte concentrations rather than native bulk ISF [8,41,42,43,44]. On the other hand, non-invasive approaches avoid physical disruption of the skin, but they remain constrained by the SC, resulting in small amount of recovered analytes. Reverse iontophoresis, for example, applies an electrical current across intact skin to transport charged and polar analytes outward by electromigration and electroosmosis [8,45]. Although this approach avoids puncturing the skin, the recovered sample is typically dilute and compositionally different from native ISF because transport depends on the analyte’s physiochemical properties and the skin barrier [46,47].

Microneedle array patches (MAPs) are arrays of micrometer-sized projection that are sufficient both for minimally invasive bypassing of the SC and for collecting ISF in its native form. MAPs are widely used to puncture the SC and create a physical pathway to access the dermis for intradermal drug delivery or dermal ISF collection [48]. Microneedle (MN) heights typically range from 25 to 2000 µm, which can puncture the SC and access the dermis yet avoid stimulating nerves, allowing the MAP application to be virtually painless [24,49,50].

Once MAPs create transient pathways across the SC, ISF extraction is governed primarily by how the pressure gradient is generated. Strategies for establishing a pressure gradient through MN-based platforms can be largely divided into passive and externally driven approaches. Passive approaches build the driving force into the device or material itself, as in capillary-driven hollow MNs or osmotic hydrogel MNs. In contrast, externally driven approaches apply positive pressure or vacuum to impose a tunable pressure gradient across the dermis, producing convective ISF transport. This distinction is important because passive mechanisms are simple and pump-free, whereas vacuum-driven convection provides a more controllable design lever under Darcy’s law.

### 2.1. Passive Generation of Pressure Gradient Using Microneedle Platforms

**Figure 3:**
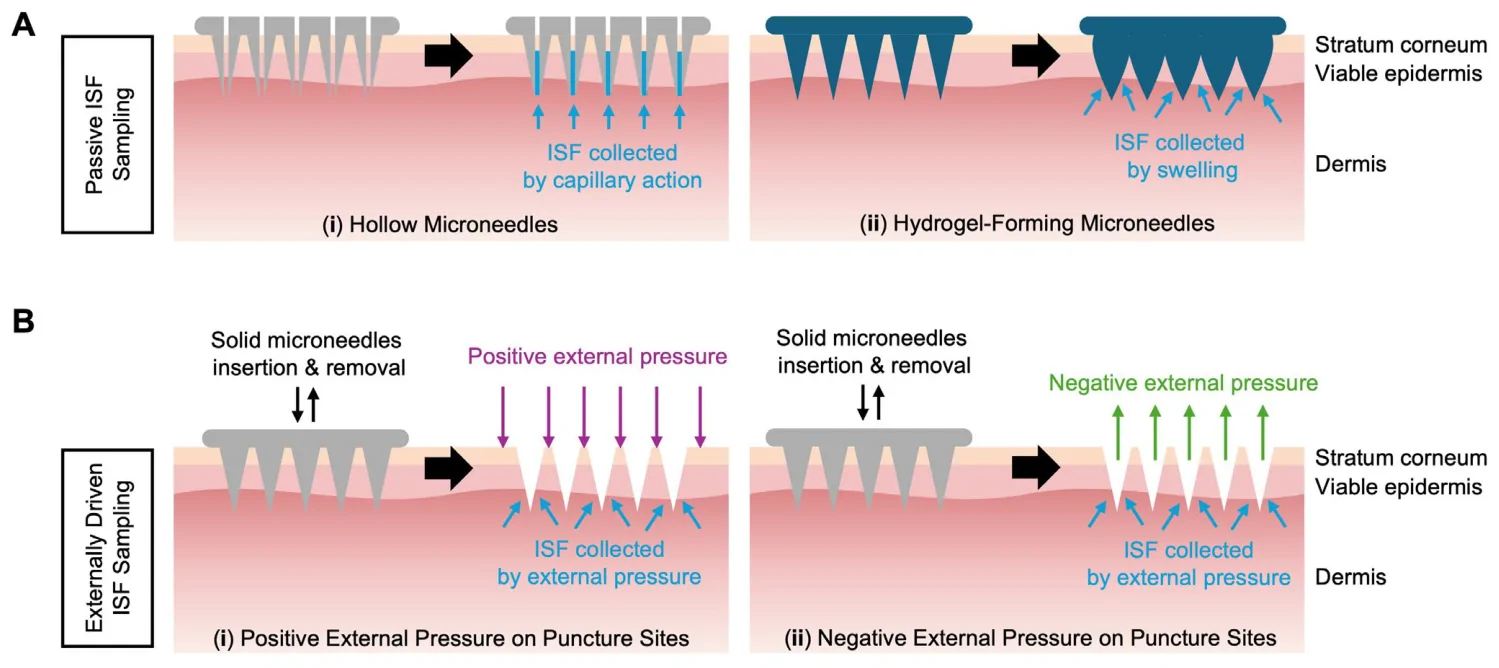
Schematic comparison of passive and externally driven microneedle-based ISF sampling mechanisms. (**A**) Passive generation of the pressure gradient: (**i**) hollow microneedles collect ISF by capillary action through narrow lumens, and (**ii**) hydrogel-forming microneedles absorb ISF through swelling of the polymer matrix. (**B**) External generation of the pressure gradient after solid microneedles create transient micropores: (**i**) positive external pressure compresses the skin around the puncture sites and drives ISF outward, whereas (**ii**) negative external pressure, such as vacuum or suction, pulls ISF from the dermis through the puncture sites toward the collection interface.

The pressure gradient can be engineered into the device itself mainly by exploiting the capillary action inside small, hydrophilic channels (Figure 3Ai). Hollow MNs have a lumen connecting the dermis to the device backing, and a sufficiently narrow, well-wetted lumen generates a passive capillary pressure that wicks ISF upward without any external pump [51]. Other pump-free hollow MN designs generate local pressure through the device geometry surrounding the MN insertion site rather than through capillary action [52]. The magnitude of the pressure gradient is set at the fabrication stage and can be tuned by adjusting the lumen geometry, raising surface wettability through hydrophilic coatings or plasma treatment, or coupling the needle to downstream microfluidic channels [53,54]. However, this same reliance on narrow lumens also makes hollow MNs susceptible to clogging by tissue debris during insertion, which can limit ISF extraction [55,56].

Hydrogel-forming MNs can passively collect the ISF through the swelling of a cross-linked polymer MNs (Figure 3Aii). These MNs, commonly built from polymer networks such as polyvinyl alcohol (PVA) or poly(ethylene glycol) diacrylate, are dry on the shelf; on skin contact, the matrix swells by imbibing dermal ISF, with the magnitude and rate of uptake set by polymer chemistry, cross-link density, and MN geometry [57,58,59]. The swelling hydrogel draws ISF into the matrix, with no external apparatus required [2,59]. After a defined sampling period, the MNs are removed from the skin, and absorbed analytes are recovered by aqueous extraction, often followed by vortexing and centrifugation [57,59]. The trade-off is that analyte recovery can be incomplete and depend on the hydrogel composition and amount of ISF uptake, complicating quantitative analysis of bulk ISF composition [2,58,59].

### 2.2. External Generation of Pressure Gradient Using Microneedle Platforms

The pressure gradient can be externally generated by the sampling device rather than by the intrinsic geometry or swelling properties of the MNs. Solid MNs can first puncture the SC and create transient micropores, after which external mechanical pressure can be applied to the puncture sites. Pressing the skin around the micropores compresses the underlying dermis and creates a spatial pressure gradient (Figure 3Bi) [19]. Alternatively, vacuum pressure can be applied over the puncture sites [6,20] or through the hollow MNs [53,54], creating a suction force to extract the ISF from the dermis to outside the skin (Figure 3Bii). Either maneuver establishes a pressure gradient between the dermis below and the collection chamber above, driving ISF outward. Because the pressure gradient is supplied externally rather than built into the needle, the magnitude and duration of the applied pressure and the vacuum can be easily tuned [6,19,20,53]. This tunability makes externally driven convection, particularly vacuum-assisted extraction, more directly aligned with Darcy’s law as a device design framework.

### 2.3. Comparative Evaluation of Passive Versus Externally Driven, Microneedle-Based ISF Sampling Mechanisms

Samant and Prausnitz (2018) systematically evaluated these mechanisms experimentally using different MN configurations on ex vivo porcine skin and compared the ISF collection volumes [22]. Capillary uptake was tested with hollow metal MNs, in which fluid entered passively by capillary action. Hydrogel MNs composed of cross-linked PVA were also tested, in which ISF uptake was measured as the matrix imbibed the fluid and swelled. Pressure-driven convection was generated in two different ways: (1) by administering a positive pressure of roughly 25 kPa by pinching the skin for 20 min; (2) by applying −85 kPa vacuum on the puncture sites for 20 min. Among these three mechanisms, pressure-driven convection, especially through vacuum more than through positive pressure, extracted the largest volumes by a wide margin, followed by capillary uptake, with the hydrogel MNs recovering the least fluid under the conditions tested. They also built a first-principle theoretical model of ISF transport with no fitted parameters, which predicted that pressure-driven convection through the micropores creates the largest pressure drop across the regions of greatest flow resistance in the dermis. The dermal pressure gradients reached 131 kPa/mm under vacuum and 77 kPa/mm under positive pressure, which remained constant throughout the 20 min experiment [22]. Together, the experimental and modeling results converge on the same design principle: vacuum applied above MAP-generated micropores both creates and sustains the largest pressure gradient across the rate-limiting barrier of the dermis and therefore recovers the most ISF among the mechanisms considered here, while remaining minimally invasive.

The systematic comparison by Samant and Prausnitz (2018) provides an experimental basis for evaluating MN-based ISF extraction mechanisms according to how the pressure gradient is generated [22]. Table 1 summarizes these mechanisms in terms of driving force, relative ISF collection yield, advantages, and limitations. This comparison highlights a key trade-off: passive approaches are free from external apparatus such as a vacuum pump, whereas externally driven approaches can generate a stronger pressure gradient and therefore collect larger ISF volume if the device–skin vacuum seal is well maintained [6,22].

In this review, we focus on vacuum-assisted MN platforms as they most directly address the central transport bottleneck identified by Darcy’s law: the need to impose a sufficient pressure gradient across the high-resistance dermal ECM [19,22]. Capillary-driven and hydrogel-forming MNs are attractive for simple, pump-free sampling, but their driving forces are largely fixed by lumen geometry, wettability, or polymer swelling capacity [2,51,56,57,58,59]. Positive external pressure can also drive convective ISF transport, but its reliance on user-applied compression can introduce variability, reduce reproducibility, and limit suitability for self-administration. In contrast, vacuum provides an externally tunable negative pressure that can be controlled by magnitude, duration, and release profile, while enabling bulk ISF extraction through micropores or hollow MN lumens.

**Figure 4:**
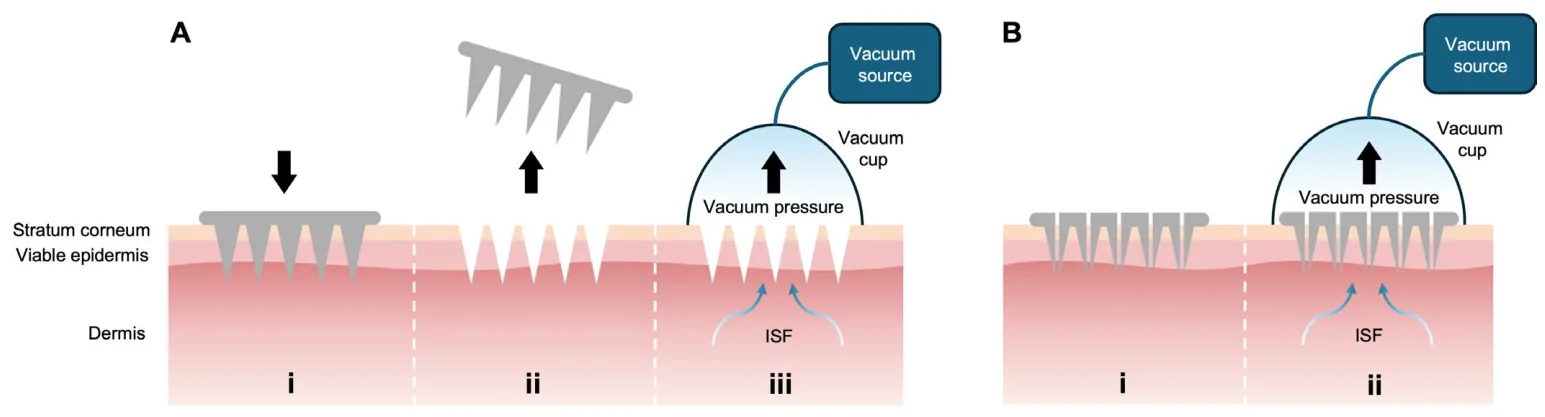
Two architectures for vacuum-assisted, microneedle-based ISF sampling. (**A**) Micropore approach, where (**i**) solid microneedles puncture the skin and (**ii**) are withdrawn, after which (**iii**) vacuum is applied above the micropores. (**B**) Hollow microneedle approach, where (**i**) MAP with hollow channels puncture the skin and (**ii**) remain inserted within the dermis during vacuum application.

Vacuum-assisted, MN-based ISF sampling mechanisms can be grouped into two architectures: micropore-based systems and hollow MN systems. In the first, solid MNs puncture the skin and are subsequently withdrawn, after which vacuum is applied above the resulting micropores to draw ISF to the skin surface (Section 3; Figure 4A). In the second, vacuum is applied directly through hollow MNs while the array remains inserted within the dermis (Section 4; Figure 4B).

## 3. Vacuum-Assisted ISF Collection Through Micropores

**Figure 5:**
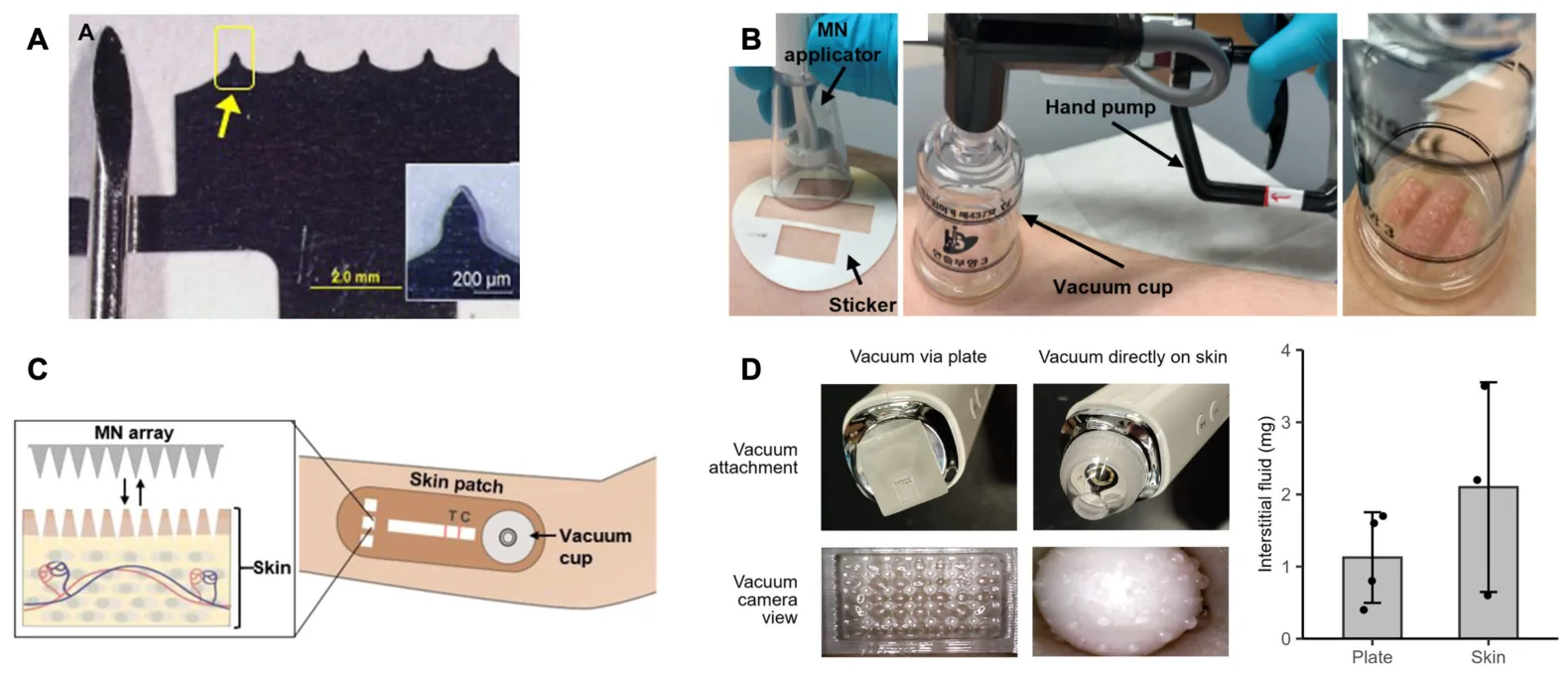
Representative studies in the micropore approach. (**A**) Stainless steel microneedle patch (**right**) shown next to a conventional lancet (**left**). Inset shows a magnified view of a single microneedle. Reproduced from Samant et al., *Science Translational Medicine*, DOI: 10.1126/scitranslmed.aaw0285 (2020), AAAS [20]. (**B**) Skin patch sticker adhered to the forearm. Microneedles are inserted using an applicator (**left**). Following microneedle insertion and withdrawal, the rigid plate is attached to the sticker, and a vacuum cup connected to a hand pump is attached (**center**). Vacuum pressure generated by the hand pump allows ISF extraction (**right**). Adapted from ref. [6] under a Creative Commons license CC BY 4.0. (**C**) Overview of the lateral flow-based skin patch, where ISF is drawn from the dermis through microneedle-generated micropores via vacuum and transported along the lateral flow immunochromatographic assay strip by surface tension. Reproduced from ref. [62] under a Creative Commons license CC BY 4.0. (**D**) Comparison of vacuum application through a collection plate and directly on the skin after microneedle insertion on human skin ex vivo. Reproduced from ref. [19] under a Creative Commons license CC By 4.0.

In the micropore approach, a MAP, typically with solid MNs, is pressed into the skin to puncture the SC and reach the upper dermis (Figure 4A). Micropore geometry is governed by MN length, tip sharpness, insertion depth, MN spacing, and the number of MNs in the MAP, which together determine the hydraulic pathway available for ISF outflow. The MAP is then withdrawn, leaving an array of transdermal micropores. Because micropores in human skin can take approximately two hours to fully recover their barrier properties in the absence of occlusion, the timing and duration of vacuum application relative to MAP insertion and withdrawal are important determinants of ISF sampling efficiency and reproducibility [6,20,60]. The kinetics of resealing depend on occlusion and may also be influenced by anatomical site and skin pigmentation [60,61]. While micropores remain open, a sealed chamber such as a vacuum cup is placed over the puncture sites, and vacuum pressure is applied to draw ISF out of the dermis, through the micropores, and onto either the skin surface or a collection element. Figure 5 summarizes representative micropore-based, vacuum-assisted ISF sampling studies.

### 3.1. Benchtop Pump-Driven Devices

The micropore approach was first demonstrated with glass MNs by Wang et al. (2005) [15]. Tapered glass needles with 15 to 40 µm tip radii and 700 to 1500 µm shaft lengths were fabricated using a thermal puller, and each tip was sealed with a Bunsen burner before use. A 1 cm^2^ area of skin was pierced with MNs to create seven to 10 micropores, either by repeated insertions of a single MN or by applying a multi-needle array once into the skin. A glass flanged bell chamber was adhered to the skin with vacuum grease and connected to a vacuum pump. Vacuum of 200 to 500 mmHg was applied for 2 to 10 min, yielding 1 to 10 µL of ISF per site in hairless anesthetized rats (*n* = 15) and six adult human volunteers. After suction, the ISF drops that had emerged on the skin surface were collected by sliding the analysis end of a glucose test strip across the puncture field. ISF glucose concentrations correlated well with paired blood glucose levels, with 95% of rat and 100% of human measurements falling within the clinically acceptable A + B region of the Clarke Error Grid [15].

The stainless-steel platform of Samant and Prausnitz (2018) followed the same puncture, withdraw, and suction workflow as Wang et al. (2005) but replaced the glass needles with stainless-steel MNs [15,22] (Figure 5A). Building on this stainless-steel MNs and external-suction approach, Samant et al. (2020) evaluated the platform in a 21-participant human metabolomics study, as well as separate caffeine pharmacokinetics and glucose pharmacodynamics studies [20]. The MAP consisted of five planar MNs, each 250 µm in length with a 10 µm tip diameter. Among the three tested MN lengths (250, 450, and 650 µm), the shortest was selected to limit penetration to the superficial dermis and thereby avoid capillary bleeding for clean, blood-free ISF samples. Micropores were created by inserting the five-MN array 20 times to generate an array of 100 micropores. After insertion, a vacuum chamber was aligned over the puncture sites, and vacuum was applied via the negative pressure cutaneous suction system, ramped from 0 to −50 kPa over about 3 min during a total vacuum application period of 20 min. In the five-participant optimization study, this method collected 2.3 ± 2.6 µL of clear ISF onto the skin surface without visual evidence of blood, which was collected by sterile medical gauze and subsequently eluted by centrifugation. Metabolomic profiling in the 21-participant study was performed by liquid chromatography mass spectrometry (LC-MS), caffeine pharmacokinetics in nine healthy adults was assessed using enzyme-linked immunosorbent assay (ELISA), and glucose pharmacodynamics in 15 children and young adults with type 1 diabetes was assessed using an Amplex Red glucose assay for ISF and a glucose analyzer for plasma [20].

### 3.2. Portable Hand Pump-Driven Devices

Jiang et al. (2024) replaced the benchtop vacuum source of the preceding micropore platforms with a portable hand pump and sealed vacuum cup placed over the puncture field [6] (Figure 5B). Solid, conical MNs with a 200 µm base diameter were fabricated from SU-8 photoresist and coated with a thin parylene layer to improve biocompatibility. Three needle heights (450, 600, and 750 µm) and two array formats (10 × 10 MNs on a 7.5 × 7.5 mm patch and 20 × 20 MNs on a 10 × 10 mm patch) were characterized. The optimal configuration was a 20 × 20 array of 450 µm needles; the 450 µm needles outperformed the 600 and 750 µm needles because every needle shared the same 200 µm base diameter, so shorter needles had a less slender taper and produced wider micropores that conducted ISF more efficiently, a result confirmed independently by pore size measurements in a Parafilm wax-membrane model. The MNs were applied to the skin three times per site with a spring-loaded applicator to generate thousands of micropores. A vacuum cup was attached over the puncture sites, and a hand pump pulled −44 kPa for 20 min. Extending suction beyond 20 min did not significantly increase collected volume and was associated with erythema, edema, or ecchymosis. After the vacuum cup was removed, the ISF that migrated to the top of the skin was collected using capillary tubes. In 28 human volunteers, a mean of 20.8 ± 19.4 µL of ISF was collected, with a mean Mankoski pain score of 1.27 ± 1.03 out of 10, and 33% of participants reported no pain and mild adverse effects such as redness and swelling that resolved within 24 h. Liquid chromatography-tandem mass spectrometry (LC-MS/MS) proteomics on paired ISF and fingerstick-derived serum from a five-volunteer subset identified 2195 distinct proteins, including 610 proteins detected in both fluids with similar abundance ratios that were identified as medically relevant using online biomarker databases (OncoMX and BIONDA). SARS-CoV-2 neutralizing antibodies (nAbs) were detected by lateral flow immunochromatographic assay (LFIA) in paired ISF and blood samples from two COVID-19 vaccinees and by ELISA in ISF from all 15 tested vaccinees, at concentrations of 81 to 618 ng mL^−1^ [6].

The same SU-8/parylene MAP and hand pump vacuum architecture was integrated with an LFIA by Wilkirson et al. (2024), placing the readout on skin and eliminating the off-device transfer step via the capillary tube that had been required previously [6,62] (Figure 5C). The 10 × 10 array of 450 µm conical MNs was kept identical to the optimized Jiang et al. (2024)’s geometry and was used to generate micropores in the skin [6]. The skin patch was then placed on the skin over the puncture sites. A vacuum cup was attached to the vacuum port of the patch, and a hand pump generated vacuum pressure for 18–20 min, drawing ISF from the dermis through the microchannels and onto the lateral flow test strip. Proof of operation used anti-tetanus toxoid immunoglobulin G (IgG) and SARS-CoV-2 nAbs as model analytes in human volunteers. The device produced a visible colorimetric readout in less than 20 min of vacuum application, making it a promising platform for rapid point-of-care diagnostic testing [62].

Hung et al. (2025) 3D-printed the devices using continuous liquid interface production (CLIP) with a dental resin, which allowed the rapid manufacturing of multiple design variants for a controlled mechanistic comparison before determining the final device design [19]. On ex vivo human abdomen skin, they tested: (i) pressure applied from above after MAP removal (the POP configuration); (ii) a needle-in variant; (iii) a vacuum variant; and (iv) a pressure-ring variant [19]. To isolate the contribution of the spatial pressure gradient to POP’s collection rate, holding the MAP and the perforated collection plate constant across all variants (7 × 5 hollow MNs with 400 µm tip diameter, mated to a plate with 35 perforations aligned to each puncture site) and varying only how the driving force was applied. Each variant was run for 5 min at a matched applied pressure (a 500 g weight, equivalent to 50 kPa, for the positive-pressure variants and −64 kPa for the vacuum variant). Finite element analysis (FEA) on a simplified dermis model was performed alongside to visualize the resulting tissue pressure distribution.

In the baseline POP variant, the MAP was withdrawn and the perforated collection plate was pressed onto the puncture sites, generating a spatial pressure gradient driving ISF flow. In the needle-in variant, the MAP remained on the skin and pressure was applied on the MAP; ISF flowed into the lumens of the hollow MNs rather than out at the puncture sites, and yield dropped because the needles still occupying the puncture sites distended the surrounding tissue and flattened the pressure gradient between each puncture site and its surroundings. In the vacuum variant, the MAP was withdrawn and −64 kPa was pulled through the perforated plate. Although FEA predicted a greater pressure gradient in the vacuum variant than POP, the experimental yields were lower. The authors attributed this discrepancy to the difficulty of maintaining individual vacuum seals across the 35 plate openings of the 7 × 5 array, so that the FEA result represented what vacuum should deliver if every seal held, whereas the experiment showed what it actually delivered when many of those seals failed. In a follow-up to this variant, vacuum was applied directly to the skin through a single aperture after both the MAP and the plate were removed (Figure 5D); the seal improved, and yields sometimes matched POP, but the lateral pressure gradient that POP depends on was lost, suggesting that vacuum suction can be competitive with POP only if reliable seals can be formed around each individual puncture site rather than across the whole field. In the pressure-ring variant, ring protrusions on the collection plate concentrated pressure in concentric rings around each puncture site rather than over the full surrounding tissue, and the MAP was lengthened to compensate for the added plate height; collection efficiency dropped because ISF was driven both toward and away from each puncture site, splitting the flow. Taken together, these comparisons identify the engineered spatial pressure gradient (high in the surrounding tissue, low at each puncture site) as the dominant driver of POP’s collection rate and identify vacuum-driven sampling as constrained primarily by the engineering of vacuum seals around each puncture site rather than by the underlying physics of pressure-driven flow through the dermal ECM [19].

### 3.3. Comparative Performance and Translational Readiness of Micropore-Based Platforms

Across these studies, the micropore approach has evolved from single-needle glass devices [15], to stainless-steel arrays [20,22], to high-density polymer arrays with portable hand pumps [6] and on-skin diagnostic patches that eliminate the off-device transfer step entirely [62]. Compared across yield, tolerability, device simplicity, analyte compatibility, and translational maturity, the micropore-based platforms show different strengths rather than a single best-performing design. Wang et al. (2005) demonstrated feasibility of the approach, but the low ISF yield and bulky benchtop vacuum setup limited broader translation [15]. Samant et al. (2020) used shorter MNs with gradual vacuum ramping to obtain clear, blood-free ISF suitable for LC-MS [20]. Jiang et al. (2024) improved yield, most substantially by making thousands of micropores instead of hundreds, while maintaining low pain scores and expanding compatibility to handheld vacuum pumps [6]. Wilkirson et al. (2024) improved device simplicity by coupling the same micropore-handheld vacuum pump to on-skin LFIA readout [62]. Overall, micropore-based platforms are relatively more clinically mature because multiple studies have demonstrated human ISF recovery with minimal pain and compatibility with downstream biomarker analysis. Their translational value depends on the intended use case, with higher-yield bulk ISF collection better suited for off-device molecular profiling and integrated readout formats better suited for targeted point-of-care testing.

The vacuum-assisted MN devices that use a micropore approach are summarized in Table 2. All platforms use the same core mechanism, in which MNs are first inserted to create transient micropores, after which vacuum is applied over the puncture sites to extract ISF. The table therefore focuses on study-specific differences in MN parameters, vacuum conditions, ISF yield, skin model, target analytes, and readout format.

## 4. Vacuum-Assisted ISF Collection Through Hollow Microneedles

In the hollow MN approach, MNs with internal channels puncture the skin and remain in place for the duration of sampling (Figure 4B). Vacuum pressure is applied through the MNs, drawing ISF directly from the dermis, through the MN lumens, and into a collection chamber or on-patch sensor. Because the MNs stay inserted, the puncture sites stay open as well, so sampling duration is less bounded by pore closure after MN removal. The continuous-needle architecture also allows the vacuum source, the fluidic channel, and the sensor to be co-located on a single wearable patch so puncture, extraction, and readout can be done in one device rather than the three-step workflow used in the micropore approach (puncture the skin with MAPs, withdraw MAPs, and apply vacuum over the puncture sites).

**Figure 6:**
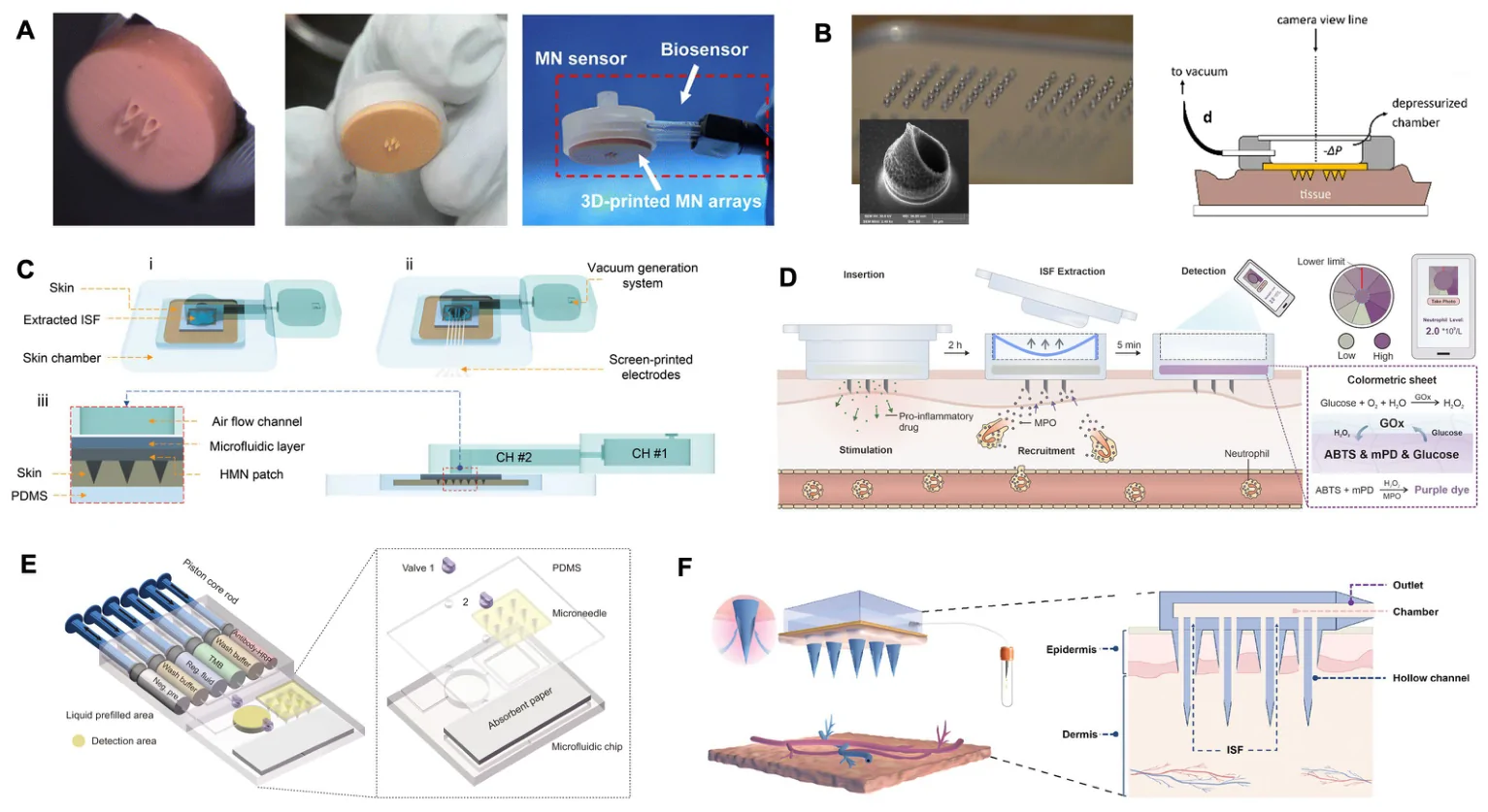
Representative studies in the hollow microneedle approach. (**A**) 3D-printed microneedle arrays with integrated biosensors for glucose, pH, and H_2_O_2_. Used with permission of the Royal Society of Chemistry, from “A 3D-Printed Microneedle Extraction System Integrated with Patterned Electrodes for Minimally Invasive Transdermal Detection,” Zhan et al., *Biomaterials Science*, *Vol. 11*, Issue 10, 2023, pp. 3737–3749 [63]; permission conveyed through Copyright Clearance Center, Inc. (**B**) Panoramic optical image of fully etched microneedle arrays. Inset shows a scanning electron microscopy (SEM) image of a single needle (**left**). Schematic illustration of how vacuum-assisted ISF extraction was performed (**right**). Used with permission of the Royal Society of Chemistry, from “Beveled Microneedles with Channel for Transdermal Injection and Sampling, Fabricated with Minimal Steps and Standard MEMS Technology,” Bagolini et al., Lab on a Chip, Vol. 25, Issue 2, 2025, pp. 201–211 [64]; permission conveyed through Copyright Clearance Center, Inc. (**C**) Schematic illustration of (**i**) ISF extraction and (**ii**) glucose and pH sensing and (**iii**) cross-sectional view of vacuum-integrated ISF extraction and sensing system (VIES). Reproduced from ref. [53] under a Creative Commons license CC BY-NC-ND 4.0. (**D**) Schematic of the button patch for neutrophil detection and quantification through a mobile phone application. Reproduced from ref. [65] under a Creative Commons license CC BY 4.0. (**E**) Schematic of the mechanism of a portable and wearable continuous immunoassay-based monitoring (CIM) device. Adapted from ref. [54] under a Creative Commons license CC BY-NC 4.0. (**F**) Schematic illustration of the vacuum tube-integrated microneedle array patch for ISF extraction. Adapted from ref. [66] under a Creative Commons license CC BY 4.0.

Devices built around this architecture vary in their vacuum source, MN material, and integrated sensor type (Figure 6). Among these design variables, the vacuum source is particularly important because it determines the degree of pressure control, device portability, user input, and compatibility with wearable or on-patch readout workflows.

### 4.1. External Pump-Driven Devices

Zhan et al. (2023) introduced a vacuum-assisted hollow MN devices in which the needle array, the vacuum delivery channel, and an electrochemical sensor were assembled into a single rigid housing [63]. The device consists of a 3D-printed 2 × 2 array of hollow conical MNs (1200 µm in length, 100 µm cylindrical channel at each tip), a customized 3D-printed shell that encases the array, and a patterned biosensor mounted on the back of the array (Figure 6A). The shell carries a narrow gas channel that connects an external vacuum source to the needle lumens and a through-hole that receives the sensor. To improve hydrophilicity, the printed surface is treated with air plasma, which lowers the water contact angle from 73.4° to 37.9°. The authors explored three vacuum levels (zero, 7.5 cmHg, and 15 cmHg) and selected 15 cmHg (~20 kPa) as the operating vacuum pressure, as it extracted the greatest volume of water. The MN array was paired with patterned electrodes built on a resin substrate textured with 20 µm-diameter, platinum-sputtered micropillars to increase the sensing surface area. Three sensor variants (glucose, pH, and H_2_O_2_) share this same micropillar electrode architecture but are functionalized differently for their respective analytes. Each sensor integrates with the MN array through a through-hole on the side of the 3D-printed shell. In diabetic Sprague-Dawley rats given an intramuscular insulin challenge, the device tracked the resulting drop in ISF glucose against a paired commercial-glucometer trace, registering a ~405 mg/dL decrease compared to ~430 mg/dL on the glucometer, with a mean detection error of 7.83 ± 9.7% and all individual points below 25% error. ISF pH and H_2_O_2_ showed only slight fluctuation, consistent with insulin selectively perturbing glucose rather than the redox or pH state [63].

Bagolini et al. (2025) replaced the 3D-printed MNs with silicon MNs fabricated using standard industrial micro-electromechanical systems (MEMS) fabrication technologies [64]. The process uses only two lithography steps and two deep reactive ion etching (DRIE) steps, with no wet etching, and produces a beveled hypodermic tip profile with the channel opening at the needle apex (Figure 6B). The resulting arrays carry 150 µm tall needles with 50 µm channel diameters, in either a high-density (H; 316 needles) or low-density (L; 136 needles) layout. The extraction setup compares two driving modes applied at the chip backside: a mechanical mass load on the chip (pressure load, P), which presses the MN array into the skin and generates pressure within the sample to drive fluid through the channels, and a vacuum line connection (vacuum, V), which evacuates the backside to draw fluid up the same channels. The four density-mode combinations (PH, PL, VH, VL) were tested in vitro on chicken-skin mockups. The pressure load conditions were increased to 500 mbar (50 kPa) over a stepped 20 min protocol and then maintained for an additional 70 min, whereas vacuum was increased from −150 to −950 mbar (−15 to −95 kPa) over 20 min. Peak yield was 2.5 µL after 90 min at 500 mbar in PH, and PL exceeded 1 µL under the same conditions. At matched low density (VL), vacuum up to −950 mbar (−15 to −95 kPa) yielded only ~0.17 µL after 20 min, and required ~150 mbar to initiate flow. Vacuum extraction was more spatially uniform across the array because it was less sensitive to skin planarity, but the authors attribute the elevated onset pressure to air leakage between the chip and the skin, confirmed by air bubbles emerging from individual channels during extraction. These same leaks, combined with incomplete penetration of the denser array under vacuum, likely contributed to the high-density vacuum condition (VH) to fail altogether [64].

### 4.2. On-Patch Vacuum-Integrated Devices

In contrast to the external vacuum sources used by Zhan et al. (2023) [63] and Bagolini et al. (2025) [64], Abbasiasl et al. (2024) integrated the vacuum source onto the patch and reported the first on-patch vacuum-integrated hollow MN device [53]. The device, named the vacuum-integrated ISF extraction and sensing system (VIES), comprises a laser-drilled polycarbonate hollow MAP, a 3D flexible microfluidic module carrying screen-printed glucose and pH electrodes, and a polydimethylsiloxane (PDMS) vacuum-generation chamber connected through two passive valves (Figure 6C). A finger press collapses the PDMS chamber, and the elastic self-recovery on release pulls negative pressure down to approximately −53 kPa through the hollow MN lumens; the second valve preserves the established vacuum across successive presses, enabling continuous and fine adjustment of the vacuum pressure. A parametric study was performed ex vivo on excised rat skin, comparing array size (3 × 3, 5 × 5, 7 × 7, and 9 × 9 arrays) at multiple vacuum pressures (−25 to −45 kPa) and durations (20 to 60 min). Increasing the array size, vacuum pressure, and duration all resulted in an increase in ISF collection. In vivo experiments on Wistar rats reproduced the same trend: per-needle volumes increased when array size increased from 3 × 3 (0.98 ± 0.1 µL of ISF) to 9 × 9 (1.89 ± 0.16 µL of ISF), when vacuum pressure increased from −30 kPa (1.03 ± 0.19 µL of ISF) to −45 kPa (1.49 ± 0.21 µL of ISF), and when vacuum duration increased from 20 min (0.23 ± 0.05 µL of ISF) to 1 h (1.18 ± 0.24 µL of ISF). ISF pH was tracked across intraperitoneal injections of pH 6.5 and pH 8.2 saline; against a commercial micro pH electrode inserted subcutaneously on the dorsal skin, the potentiometric on-patch sensor agreed within a detection error of 3.18 ± 1.0% over a 3 h period. The VIES also tracked ISF glucose responses to intraperitoneal glucose and insulin/glucose challenges, with the amperometric ISF signal following the same trend as paired tail-vein blood glucose measured by a commercial glucometer. The authors report successful in vivo detection of both ISF glucose and pH, supporting the feasibility of continuous MN-based ISF sampling and electrochemical sensing without external pumping equipment [53].

Gao et al. (2025) developed a simplified on-patch vacuum system and paired it with a multiplexed colorimetric immunoassay readout [67]. The device, called a MN sensor for real-time inflammation monitoring (MS-RTIM), consists of a 2 × 2 stainless-steel hollow MN array at the base of a sealed vacuum chamber capped by a compliant PDMS film; pre-pressing the film with a finger and releasing it generates the negative pressure that draws ISF up through the needle channels. The geometry was chosen after a parametric sweep on rat skin, yielding ~1.30 ± 0.20 µL of ISF per needle after 5 min of extraction. The chamber floor carries a flower-shaped nitrocellulose immunochip with a central sample zone and multiple peripheral test zones, each pre-coated with a different capture antibody. After the ISF wets the chip and a small volume of running buffer flushes the analytes outward, a mixed biotin-detection-antibody/avidin-horseradish peroxidase (HRP) cocktail and a 3,3′,5,5′-tetramethylbenzidine (TMB) substrate are added in sequence, and HRP-catalyzed oxidation of TMB develops a naked-eye blue color on each test zone in parallel. Four inflammatory proteins—Interleukin (IL)-6, IL-1β, tumor necrosis factor (TNF)-α, and C-reactive protein (CRP)—are detected simultaneously from a single extraction, with linear ranges of 5–500 pg/mL for IL-6, IL-1 β, and TNF-α, and 0.25–50 ng/mL for CRP. In vivo, MS-RTIM was applied to a rat model of acute systemic inflammation induced by lipopolysaccharide injection (15 mg/kg) and read out at 0, 4, 12, and 24 h; the colorimetric trends tracked the kinetics of paired serum ELISA references for all four targets, with early IL-6, IL-1β, and TNF-α surges at 4 h and a later CRP peak at 12 h. The micropores recovered within 1 h of device removal, and hematoxylin and eosin (H&E) sections taken 24 h after removal showed no significant tissue damage [67].

A similar pre-pressed PDMS vacuum chamber architecture to that of Gao et al. (2025) [67] was also used by Lu et al. (2025), with the colorimetric immunochip replaced by a hydrogel-based enzymatic sensor for neutrophil-derived myeloperoxidase (MPO) [65]. The button patch consists of a 3 × 3 array of laser-cut stainless-steel hollow MNs (950 µm needle height, 150 µm inner channel diameter) at the base of a sealed vacuum chamber capped by a PDMS soft film; pre-pressing the film with the cover stores negative pressure, and removing the cover initiates ISF extraction through the needle channels (Figure 6D). Inside the chamber, a lyophilized two-layer gelatin methacrylate (GelMA) hydrogel sheet serves as the sensor; when ISF wets the sheet, glucose oxidase (GOx) oxidation of glucose generates H_2_O_2_ in situ, and neutrophil-derived MPO in the ISF yields a purple chromophore on the observation window, quantified via a mobile phone application against a built-in colorimetric card. In vivo, the device was applied to rat dorsal skin and held for 2 h to allow local neutrophil recruitment by 100 µg preloaded phorbol 12-myristate 13-acetate; the cap was then removed, and ~5 µL of ISF was drawn into the hydrogel sheet within 5 min. At 2 h after phorbol 12-myristate 13-acetate stimulation, ISF MPO levels in normal rats were ~8.3-fold higher than those in cyclophosphamide-induced neutropenic rats, with corresponding neutrophil recruitment confirmed by H&E staining, whereas cyclophosphamide-induced neutropenic rats showed negligible MPO changes and minimal neutrophil infiltration. In human subjects, only device safety was validated; the sterilized patch was applied to the skin for 2 h, after which the micropores closed within 10 min and the skin fully recovered within 5 h [65].

Chen et al. (2025) addressed a constraint common to antibody-based assays: the lack of sensor reusability and regeneration after antigen capture [54]. The continuous immunoassay-based monitoring (CIM) device retains the hollow MN and negative pressure, as well as a regenerable readout that supports at least 10 consecutive C-peptide detections on a single chip with 0.1% signal attenuation. The MAP is 3D-printed as a 6 × 6 array of conical bichannel hollow MNs (650 µm tip height, 150 µm channel diameter). The device consists of three components (MN part, reaction chamber, and injection part), fed by five prefilled syringes carrying a detection antibody conjugated to HRP, wash buffer, TMB substrate, a glycine–hydrochloric acid regeneration solution, and a second wash buffer, plus a sixth syringe used to pull the vacuum (Figure 6E). C-peptide in the extracted ISF was quantified by a regenerable double-antibody sandwich assay (biotin–streptavidin-immobilized antibody A, HRP-labeled antibody B, TMB chromogenic readout). Color was read out by a smartphone app trained on red, green, and blue (RGB) features. In vivo, ~62.9 ± 4.8 µL of ISF was drawn from rabbit ear skin within 5 min and C-peptide was tracked continuously over a 36 h session across four elution–regeneration cycles, with less than 5% deviation from non-eluted controls. In mice in vivo, the CIM device discriminated ISF C-peptide concentrations in type 1 diabetic, type 2 diabetic, and normal mice [54].

### 4.3. Pre-Evacuated Vacuum Tube-Integrated Devices

Xie et al. (2024) replaced the vacuum source with a pre-evacuated external vacuum tube (VT) connected to the patch through a flexible hose, no longer requiring active user input during sampling [66]. The MAP was 3D-printed in biocompatible polymethylacrylate (PMA) resin on a micro-nano printer, with a 10 × 10 array of bichannel hollow needles, each 1000 µm tall with 250 µm diameter channels (Figure 6F). The VT supplied a negative pressure of only 75 Pa, orders of magnitude below the kPa-level negative pressures generated by vacuum cups, yet sufficient to drive ISF through the lumens with negligible skin invasion. In live New Zealand rabbit ears, the device collected 18.42 ± 1.02 µL of ISF in 5 min. By preloading the hose with different test papers, the device read out a range of analytes directly: a commercial lateral flow test strip detected dexamethasone, a luminol-based chemiluminescent paper detected lactate, and colorimetric enzyme and pH-indicator papers detected glucose and pH. All four readouts were demonstrated on live rabbit ear ISF. The colorimetric glucose reading also tracked the rise and fall of ISF glucose after the rabbit was given a sugar drink and later an insulin injection, matching concomitant rabbit ear vein blood glucose measurements obtained with a commercial glucometer. No human extraction was performed [66].

Xie et al. extended their VT platform by making it wearable and adding a convolutional neural network (CNN) classifier for smartphone images of the colorimetric readout [68]. The 3D-printed PMA MAP and pre-evacuated VT were retained and made wearable by a silicone wrist or upper-arm belt, yielding the wearable MN array-based colorimetric (WMNC) sensor. Separate nitrocellulose sensing papers were inserted near the outlet port, one for sodium ion and one for uric acid. A CNN classified each readout as low, normal, or high with ~99% accuracy, mitigating the effects of variable ambient lighting. In live Sprague-Dawley rats, the device drew ~14 µL of dorsal-skin ISF within 5 min, and ISF sodium and uric acid tracked paired blood measurements during a desmopressin acetate (DDAVP) challenge. Long-term DDAVP administration decreased both sodium and uric acid levels; the uric acid concentration was sufficiently low that the commercial uric acid meter read below its lower limit, consistent with the syndrome of inappropriate antidiuretic hormone secretion (SIADH)-like phenotype the device was designed to flag. No human ISF extraction was reported [68].

Iftikhar et al. (2026) extended the VT-integrated hollow MN array patch (HMNsAP) of Xie et al. (2024) with a flap microvalve and an electrochemical sensor for uric acid [66,68,69]. The flexible flap, 3D-printed in place above the chamber outlet, opens under vacuum and reseats under backflow, preventing reagents in the sensing chamber from leaking into the skin. The chamber houses a screen-printed electrode (SPE) whose working electrode is modified with a metal organic framework (MOF)-on-MOF nanocomposite, named HKUST-1@Eu-MOF, paired with an Ag/AgCl reference and a platinum counter electrode. By differential pulse voltammetry, the sensor covered 0.02–600 µM uric acid with a 20 nM detection limit and 1.68% relative standard deviation across ten electrodes. Ex vivo testing on excised rat dorsal skin pre-soaked in 200, 450, and 570 µM uric acid gave 98.6–100.4% recovery. In vivo skin penetration study was done on BALB/c rats, in which micropores from the 10 × 10 array faded within ~20 min after patch removal. No in vivo ISF was extracted or analyzed [69].

### 4.4. Comparative Performance and Translational Readiness of Hollow MN-Based Platforms

Hollow MN devices for vacuum-assisted ISF collection have followed three architectures: external pump-driven systems, on-patch vacuum-integrated systems, and pre-evacuated VT-integrated systems. External pump-driven setups couple the MAP to a benchtop vacuum or vacuum line, providing direct control over vacuum magnitude and duration, making them useful for mechanistic studies of pressure-driven ISF extraction and hollow MN geometry [63,64]. However, the need for tubing, vacuum lines, and external control hardware can limit portability, self-administration, and long-term wearable use. Therefore, external pump-driven systems are best viewed as controlled engineering platforms rather than fully translatable clinical or at-home collection devices.

On-patch designs move the vacuum generation onto the patch itself, typically through a finger-actuated PDMS chamber that seals after release [53,65,67]. Compared with external pump-driven systems, these platforms are more portable and better suited for wearable or point-of-care operation because puncture, vacuum generation, fluidic transfer, and sensing can be integrated into a single device. Their translational strength, however, lies primarily in on-patch readout of selected biomarkers rather than in recovery of bulk ISF for off-device analysis. In most cases, ISF extraction serves as an upstream step to deliver analytes to an integrated electrochemical, colorimetric, or immunoassay readout, rather than to capture the full recovered fluid volume as a standalone sample. Therefore, while on-patch systems are highly attractive for wearable monitoring, they may be less suitable for workflows that require off-device multi-analyte, multi-omic, or centralized laboratory analysis. In addition, their performance can depend on chamber mechanics, user actuation, and maintenance of a reliable device–skin seal. As a result, on-patch systems reduce equipment dependence but may introduce variability in the applied pressure gradient, particularly during self-administration or extended wear.

Pre-evacuated VT designs use a vacuum tube as the negative-pressure source, removing the need for continuous active pumping and active user input [66,68,69]. This architecture is translationally attractive because it simplifies the overall setup and reduces the need for active user operation, making it potentially better suited for at-home or point-of-care translation. A major translational advantage of this design is its readout flexibility; Xie et al. (2024) demonstrated that the ISF collected using their VT-integrated MAP could be transferred from the hose onto a commercial glucometer testing pad for prompt glucose readout, while also showing built-in sensing by preloading the hose with analyte-specific test papers [66]. However, VT-integrated systems still require stable vacuum retention before use and reliable coupling between the pre-evacuated tube and the hollow MN patch. Thus, the main translational advantage of VT-integrated systems is simplified, user-friendly vacuum generation with compatibility for both off-patch and built-in point-of-care readout, whereas a remaining limitation is the lack of demonstrated reproducible human ISF collection.

Beyond differences in vacuum source, hollow MN platforms share several limitations that remain important for translation. First, fabrication can be more complex than in micropore-based systems because needle sharpness, lumen patency, mechanical strength, and reliable insertion depth must be controlled simultaneously. Second, lumen clogging remains a recognized design concern. Abbassidal et al. (2024) addressed this risk by using side-opening hollow MN geometries, reasoning that side openings may reduce tissue clogging during insertion compared with beveled or tip-facing openings [53]. Third, ISF recovery efficiency may be limited by dead volume within the lumen, hose, or chamber, adsorption to internal surfaces, and incomplete transfer of ISF from the hollow MN array to the collection or sensing element. Finally, human and long-term wearable validation remain limited. Most in vivo studies have been conducted in rodent or rabbit models; only Lu et al. (2025) tested the patch on humans, but only for safety, not ISF extraction [65]. The device parameters, vacuum protocols, and collection performance of the studies discussed are summarized in Table 3.

## 5. Discussion

ISF flow in the dermis described by Darcy’s law introduces two levers for designing an ISF sampling device: hydraulic conductivity and pressure gradient. For clinical translation, increasing the pressure gradient is the more practical and less disruptive lever because strategies that modify hydraulic conductivity, such as tissue pretreatment or hydration, may improve ISF recovery but can also perturb the native sampling environment and introduce additional variability. Vacuum-assisted MN platforms are therefore particularly attractive: MNs bypass the SC with minimal skin invasion, while vacuum provides a tunable pressure gradient that can directly draw ISF into a collection or sensing element. Vacuum can also be generated by several device-compatible sources, including external pumps [15,20,63,64], portable hand pumps [6,62], battery-powered cosmetic vacuum devices [19], and wearable mechanical or microfluidic reservoirs such as PDMS bellows [53], soft-film chambers [65,67], and pre-evacuated vacuum tubes [66,68,69]. These platforms therefore provide a practical foundation for reproducible, user-friendly ISF sampling in both off-device analysis and device-integrated sensing applications.

The two architectures examined in this review differ in how the pressure gradient is coupled to the skin and what analytical endpoint they best support. Micropore-based systems apply vacuum over transient puncture sites after MAP removal, making them generally better suited for recovering bulk ISF for off-device analysis. Hollow MN-based systems retain the needles in the skin and apply vacuum through internal lumens, making them well suited for integrated sensing and wearable monitoring. Table 4 summarizes the key architectural, analytical, and translational differences between micropore-based and hollow MN-based vacuum-assisted ISF sampling platforms.

This comparison highlights that the two architectures should be optimized for different clinical use cases and biomarker analyses. For off-device liquid biopsy, the major requirements are sufficient ISF volume, preserved biomarker concentration and integrity, minimal dilution or contamination, low sample transfer loss, and compatibility with downstream workflows such as LC-MS/MS, immunoassays, proteomics, metabolomics, and potential nucleic acid analysis. These requirements favor micropore-based systems, which are designed to recover ISF as a bulk, native sample and have more advanced human validation to date. In contrast, on-patch targeted sensing does not necessarily require large bulk ISF volumes; instead, it requires reproducible ISF delivery to the sensor, stable calibration, drift control, biofouling resistance, reagent stability, and reliable signal readout. These requirements favor hollow MN-based systems, which can integrate skin access, ISF transport, and targeted sensing within a single wearable platform.

For real-time monitoring, hollow MN platforms offer a natural route for biosensor integration, but each sensing modality introduces specific limitations. Electrochemical sensors are attractive for miniaturized continuous monitoring but remain sensitive to drift, interference, and biofouling [70,71]. Colorimetric and chemiluminescent sensors can support visual or smartphone-readable assays but require stable reagents and reliable optical readout [65,67,68]. Multiplexed sensors are promising for targeted biomarker panels, but their performance depends on calibration, cross-reactivity control, reagent stability, and signal normalization [71,72].

Both architectures nonetheless share a physical bottleneck that becomes visible in head-to-head comparisons. Hung et al. (2025) showed that, at matched needle geometry and skin condition, an open vacuum cup over a finite micropore field collected 1.9 to 4 times less ISF than a pressure-load configuration, a difference the authors attributed to the difficulty of sustaining a tight seal at every puncture site simultaneously across an array [19]. The MEMS-fabricated hollow silicon MNs of Bagolini et al. (2025) showed the same observation in the hollow MN approach; vacuum extraction collected 0.17 µL, compared with 1.1 µL under the equivalent pressure-load configuration, and high-density vacuum experiments failed altogether because of air leaks between the chip and the skin, combined with incomplete penetration, skin compaction, and bending [64]. The convergence of these two findings across different architectures suggests that the device-to-skin seal under negative pressure is a shared limiting factor. One practical way to improve vacuum seal integrity is to use an adhesive-backed interface, as Wilkirson et al. (2024) reported that the adhesive backing of their patch created an airtight seal with the skin and allowed vacuum pressure to be maintained throughout the test [62].

In addition to device–skin seal integrity, user and skin variabilities remain important translational bottlenecks. User-dependent factors, such as MAP insertion force, device placement, and application duration, could directly affect dermal access and keeping the vacuum seal throughout the collection, hugely affecting ISF yield, especially in self-administered or at-home settings [19,73,74,75,76]. Skin-dependent factors are also important, because anatomical site, local curvature, hair, wrinkles, skin thickness, age, and skin disease may influence MAP insertion, vacuum sealing, and fluid recovery [75,77,78,79,80]. These variables are not necessarily problems that can be fully solved by device engineering alone, but they define the robustness that future devices must achieve. Standardized application sites, user-friendly applicators, conformal sealing interfaces, and validation across representative users and skin conditions will therefore be essential for clinical translation.

Overall, micropore-based systems are currently supported by stronger in vivo human data for pain tolerability and compatibility of the collected ISF with off-device ISF analysis, whereas hollow MN-based systems may be more suitable for future wearable and real-time monitoring applications once human ISF extraction, reproducibility, and sensor stability are further validated. Regulatory considerations should also be aligned with the intended clinical function of the platform, as well as target biomarker, disease context, and intended user setting. Future device development should therefore be guided by the intended clinical endpoint, with performance evaluated not only by ISF volume but also by sample integrity, reproducibility, user-independent operation, device–skin vacuum seal robustness, and compatibility with the intended downstream analysis or sensing workflow.

## 6. Future Direction

**Figure 7:**
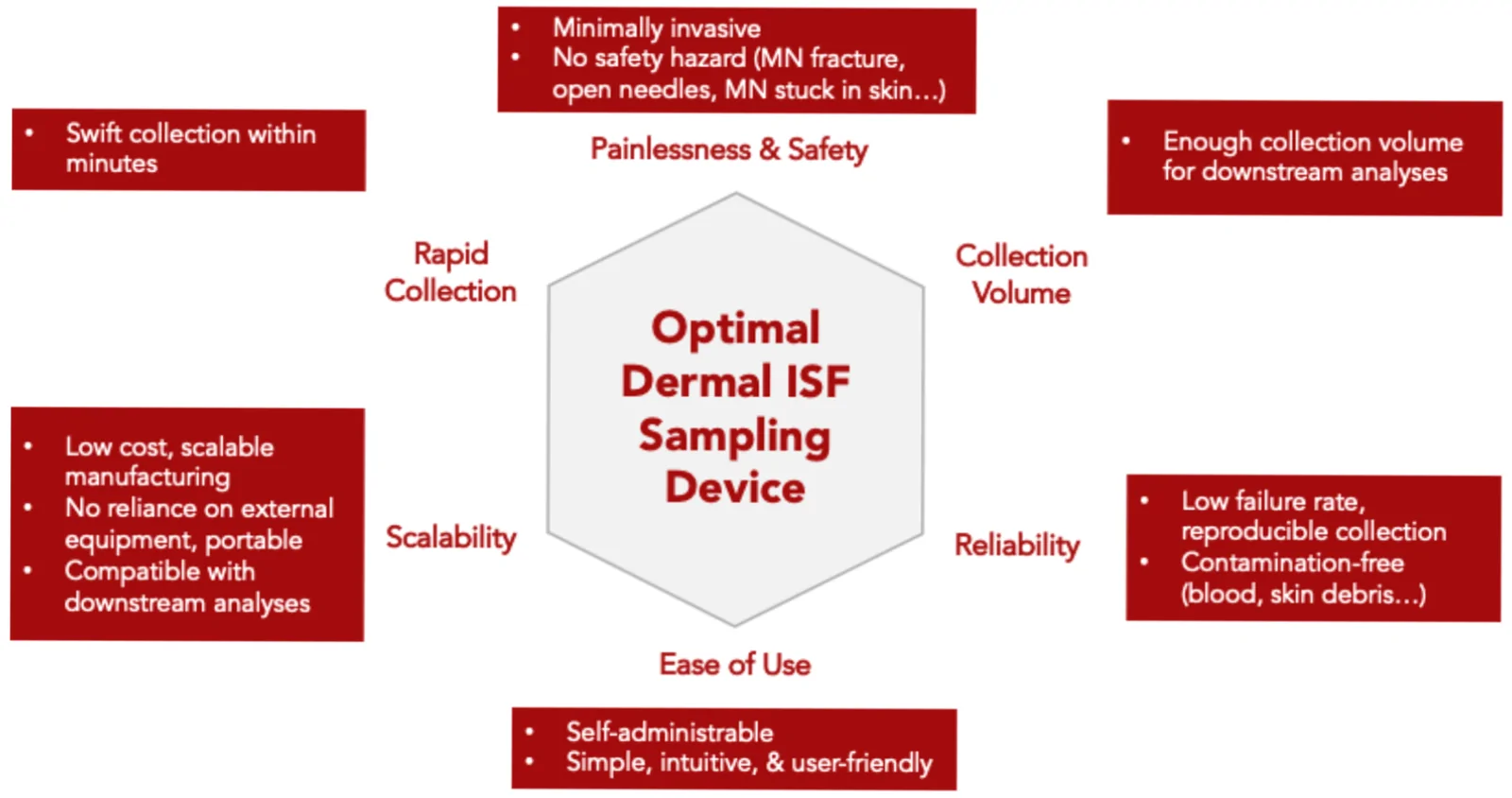
Criteria for optimal dermal ISF sampling device.

An ideal vacuum-assisted MN device suitable for both clinical point-of-care use and at-home self-administration must simultaneously satisfy six design criteria, summarized in Figure 7.

The first is painlessness and safety, which requires that the MNs minimize stimulation of dermal nerves and show mechanical integrity sufficient to resist fracture, deflection, or retention of broken tips in the skin. Practically, pain should be benchmarked against existing blood sampling methods. For clinical or at-home use, ISF collection should ideally produce pain scores comparable to or lower than finger-prick blood collection or phlebotomy, while also minimizing erythema, bleeding, irritation, and other adverse skin responses. The second is collection speed, which requires ISF collections on the order of minutes rather than hours, consistent with the 5 to 20 min protocols reported across both architectures reviewed here [6,20,54,62,65,66,67,68,69]. The third is collection volume, which must be matched to the intended downstream analysis. Although some studies succeeded in performing LC-MS or colorimetric assays using single-digit microliters of ISF [20,65], collecting tens-of-microliters of ISF would be preferable for repeat measurements and broader analytical flexibility.

The fourth criterion is reliability, encompassing reproducible ISF yield per collection and per subject, samples free of blood and skin debris, and a robust device–skin vacuum seal. Reliability should be quantified by successful collection rate, seal failure rate, blood contamination rate, and inter- and intra-user variability in ISF yield. A zero-failure device would be ideal; ISF sampling devices should be capable of reproducible collection across users, skin sites, and repeated applications. The fifth is ease of use, which requires that applying the device should not require training or medical expertise; a device should be user- and beginner-friendly, preferably self-administrable. Ease of use should be quantified using validated usability tools such as the system usability scale (SUS) [81], together with objective measures, including independent-use success rate, number of user steps, completion time, need for operator assistance, and user error rate. Ideally, untrained users should be able to complete ISF collection using only written or video instructions. The sixth is scalability, which requires manufacturing routes compatible with high-volume production at low unit cost, portability without external pumps or wired equipment, and compatibility with the intended downstream analytical pipeline. For clinical translation, the total cost and logistical burden of ISF collection should be competitive with existing sampling workflows, including clinic-based blood draws and emerging at-home blood collection devices. No platform reviewed here satisfies all six criteria simultaneously, and the trajectory of the field will be determined by how rapidly the remaining gaps are closed.

Translating a vacuum-assisted, MN-based ISF sampling platform into a device that meets these criteria will require consolidating the engineering elements demonstrated across the studies into a single miniaturized format. To enable accessible and decentralized liquid biopsy for more frequent disease monitoring, the field should move toward self-administrable MAP platforms that are user-friendly enough to operate without trained personnel or specialized equipment.

In the short term, the most immediate hardware priority is replacement of the external vacuum sources that still dominate the micropore architecture with the on-patch vacuum sources already established in the hollow MN architecture, including finger-pressed PDMS bellows [53], pre-pressed soft-film chambers [65,67], and pre-evacuated vacuum tubes [66,68,69]. Equally critical is the resolution of vacuum-seal integrity at the device–skin interface, which Hung et al. (2025) and Bagolini et al. (2025) identified as a limiting factor [19,64]. Compliant interface materials, gasketed apertures, and skin-friendly adhesives offer plausible engineering routes to that end. Mechanical robustness, sterility, and biocompatibility under prolonged or repeated skin contact must also be addressed at the design stage.

In the mid-term, future studies should move beyond proof-of-concept demonstrations and validate performance across healthy human volunteers and skin conditions. Key validation goals include reproducible ISF yield across users, skin sites, age groups, and repeated applications; low vacuum-seal failure and blood contamination rates; acceptable pain and skin tolerability; and preserved analyte recovery during collection, transfer, and storage or on-patch sensing. These studies should also benchmark usability in untrained users and determine whether ISF collection can be completed using only written or video instructions, without trained personnel or specialized equipment.

In the long term, the appropriate clinical translation pathway depends on the intended use case. When the goal is recovery of ISF as a whole biofluid for downstream multi-omic analysis, the platform should terminate in a sealed collection container (for example, a microcentrifuge tube or a pre-evacuated collection tube), taking advantage of the fact that ISF, unlike whole blood, clots less [22]. This will allow samples to be self-collected at home and shipped to a centralized laboratory for analysis. When the goal is on-patch detection of a specific target analyte, integrated hollow MN devices that perform ISF sampling and readout on a single patch are the natural endpoint, as illustrated by C-peptide [54], neutrophil-derived MPO [65], inflammatory cytokine [67], and uric acid [68,69] platforms. These two pathways impose different engineering and translational constraints and should be developed in parallel rather than treated as competing alternatives.

Closing these engineering gaps in parallel would position vacuum-assisted MN devices as a viable platform for routine ISF-based diagnostics outside the centralized laboratory.

## 7. Conclusions

This review framed dermal ISF sampling through Darcy’s law, identifying the pressure gradient as the most directly controllable device-level variable and one that can be adjusted without directly modifying the native dermal matrix. Vacuum applied through MN-generated micropores was introduced as the effective driving mechanism for generating pressure gradient and increasing ISF collection. Two architectures by which vacuum is coupled to a MAP were surveyed and critically compared in this review. The micropore approach inserts a MAP, withdraws it, and applies vacuum over the resulting micropores. The hollow MN approach keeps the MAP in the skin and pulls vacuum through the lumens. For each architecture, representative studies were summarized, and the design evolution from early demonstrations to the most recent platforms was traced.

Direct comparison highlighted the structural difference that distinguishes the two architectures, which translates into asymmetric trade-offs across sampling continuity, analytical breadth, and operational complexity. Both architectures, however, shared a common bottleneck: achieving a reliable vacuum seal at the device–skin interface, which has been reported to limit ISF collection volumes substantially below theoretical predictions. Broader adoption of vacuum-assisted MN platforms will also require replacing external vacuum sources with integrated on-patch vacuum reservoirs, improving reproducible ISF yield across users and skin sites, enabling user-friendly operation, and achieving scalable device fabrication. The clinical translation of vacuum-assisted, MN-based ISF sampling was further mapped, with the micropore approach better suited to in-clinic, single time-point liquid biopsy applications, and the hollow MN approach more compatible with self-administered or wearable continuous monitoring.

Finally, six design criteria for an ideal point-of-care ISF sampling device were pro- posed, together with the engineering advances needed for vacuum-assisted MN platforms to support both clinical and at-home use.

## Figures and Tables

**Table 1:** Comparison of passive and externally driven, microneedle-based ISF sampling mechanisms.

	Capillary-Driven Hollow MNs	Hydrogel-Forming MNs	Positive External Pressure	Negative External Pressure
Category of Pressure Gradient Generation	Passive	Passive	Externally driven	Externally driven
Driving Force	Capillary pressure within narrow lumens	Swelling of dry polymer matrix	Applied compression around MN-created micropores	Vacuum applied over MN-created micropores
Relative ISF Collection Volume [22]	Moderate	Low	High	Highest
Main Advantages	Pump-free operation; direct collection of ISF;	Pump-free operation; tunable hydrogel swelling	Tunable pressure gradient; direct collection of ISF	Tunable pressure gradient; direct collection of ISF
Main Limitations	Surface treatment often required; prone to lumen clogging	Incomplete analyte recovery; extraction/elution required during downstream processing	User-dependent compression, not suitable for self-administration	Requires external vacuum; risk of device–skin vacuum seal leakage

**Table 2:** Device parameters and collection performance of vacuum-assisted microneedle ISF sampling devices that use a micropore approach.

Ref.	MN Material and Geometry	MAP Insertion Format	Vacuum Source	Vacuum Pressure	Vacuum Duration	ISF Collected	Skin Model	Analyte/ Readout
Wang et al. (2005) [15]	Glass; MN height = 700–1500 µm; single MN	Repeated insertions to make 7–10 micropores in 1 cm^2^ area of skin	External pump via glass bell chamber	200–500 mmHg (~26.7–66.7 kPa)	2–20 min	1–10 µL	In vivo rat (*n* = 15); in vivo human (*n* = 6)	Glucose/ Off-device glucose test strip
Samant et al. (2020) [20]	Stainless steel; MN height = 250 µm; 5 MNs per patch	20 insertions to make 100 micropores	External pump	−50 kPa	20 min	2.3 ± 2.6 µL	In vivo human; multiple cohorts	Metabolomics, caffeine pharmacokinetics, glucose pharmacodynamics/ off-device LC-MS, ELISA, glucose assays
Jiang et al. (2024) [6]	SU-8 + parylene; MN height = 450 µm; 100–400 MNs per patch	Repeated insertions to make thousands of micropores	Hand pump	−44 kPa	25 min (vacuum cup removed after 20 min)	20.8 ± 19.4 µL	In vivo human (*n* = 28)	Proteins, SARS-CoV-2 nAbs/ off-device LC-MS/MS, LFIA, and ELISA
Wilkirson et al. (2024) [62]	SU-8 + parylene; MN height = 450 µm; 100 MNs per patch	1 insertion	Hand pump	Not reported	18–20 min	14.8 ± 2.9 μL	In vivo human (*n* = 4)	Anti-tetanus toxoid IgG, SARS-CoV-2 nAbs/ on-patch LFIA
Hung et al. (2025) [19] *	KeySplint resins; MN height = 2200 µm, exposed height = 1100 µm when mated with the collection plate; 35 MNs per patch	1 insertion	Facial vacuum through custom vacuum adapter	−64 kPa	5 min	1.125 ± 0.63 mg	Ex vivo human	N/A

* Parameters correspond to the vacuum variant from the mechanistic comparison in Hung et al. (2025), not their final device (puncture-out-and-press, POP) [19]. The POP uses positive mechanical pressure; the vacuum variant is included here for comparison within the vacuum-assisted micropore architecture.

**Table 3:** Device parameters and collection performance of vacuum-assisted microneedle ISF sampling devices operating on a hollow MN mechanism.

Ref.	MN Material and Geometry	Vacuum Source	Vacuum Pressure	Vacuum Duration	ISF Collected In Vivo	In Vivo Model	Analyte/Readout
Zhan et al. (2023) [63]	Acrylate-based photopolymer, air plasma-treated; MN height = 1200 µm; 4 MNs per patch	External air extraction device via narrow gas channel on the shell	15 cmHg (~20 kPa)	Not reported	Not reported	Rat	Glucose, pH, H_2_O_2_/on-patch patterned Pt micropillar electrodes
Bagolini et al. (2025) [64]	Silicon; MN height = 150 µm; 136–316 MNs per patch	Facility vacuum line connected through 500 mL vacuum lung and needle valve	−0.15 to −0.95 bar (−15 to −95 kPa)	20 min	Not reported	N/A	N/A
Abbasiasl et al. (2024) [53]	Laser-drilled polycarbonate; MN height = 1500 µm; 9–81 MNs per patch	On-patch finger-pressed PDMS actuator with two passive check valves	−25 to −40 kPa	20 min to 1 h	Up to 17.03 ± 1.40 µL h^−1^	Rat	Glucose and pH/ on-patch screen-printed electrochemical sensors
Gao et al. (2025) [67]	Stainless steel; MN height = 1700 µm; 4 MNs per patch	On-patch pre-pressed PDMS soft-film vacuum chamber	Not reported	5 min	Not reported	Rat	IL-6, IL-1β, TNF-α, CRP/ on-patch colorimetric immunochip read
Lu et al. (2025) [65]	Laser-cut stainless steel; MN height = 950 µm; 9 MNs per patch	On-patch pre-pressed PDMS soft-film vacuum chamber	Not reported	5 min	~5 μL	Rat	Neutrophil-derived MPO/on-patch colorimetric hydrogel sheet
Chen et al. (2025) [54]	PMA resin; MN height = 650 µm; 36 MNs per patch	On-patch syringe-driven negative pressure	25, 50, 66, and 75 kPa tested to observe safety	5 min	62.9 ± 4.8 µL	Mouse, rabbit	C-peptide/on-patch regenerable double-antibody sandwich immunoassay
Xie et al. (2024) [66]	PMA resin; MN height = 1000 µm; 100 MNs per patch	Pre-evacuated vacuum tube (VT) connected via flexible hose	75 Pa	5 min	18.42 ± 1.02 µL	Rabbit	Glucose, lactate, dexamethasone, pH/off-device commercial glucometer and on-patch sensing papers preloaded inside the hose
Xie et al. (2025) [68]	PMA resin; MN height = 1000 µm; 100 MNs per patch	Pre-evacuated VT connected via flexible hose	Not reported	5 min	~14 µL	Rat	Sodium and uric acid/on-patch nitrocellulose strip inside the hose
Iftikhar et al. (2026) [69]	Biocompatible resin; MN height = 1200 µm; 100 MNs per patch	Pre-evacuated VT via hose; integrated flap microvalve inside the chamber	Not reported	Not reported	Not reported	Rat	Uric acid/on-patch DPV on HKUST-1@Eu-MOF-modified, screen-printed electrode

**Table 4:** Comparison of micropore-based and hollow MN-based vacuum-assisted ISF sampling architectures.

	Micropore-Based	Hollow MN-Based
Skin Access Mechanism	MAP creates transient micropores and is removed before ISF collection.	Hollow MNs remain inserted during ISF collection.
Vacuum Application Site	Applied over the puncture sites on the skin.	Applied through internal MN lumens.
Primary Output	Recovered bulk ISF sample.	Recovered ISF or integrated/on-patch sensor readout.
Main Translational Niche	Bulk ISF sampling for off-device liquid biopsy analysis.	Wearable targeted sensing and repeated or continuous monitoring.
Analytical Compatibility	Better suited for off-device analysis because recovered ISF can be transferred to external laboratory workflows, including LC-MS/MS, immunoassays, proteomics, metabolomics, and potential genomic/transcriptomic analysis. Integrated biosensing is possible but usually requires an additional assay zone or transfer step.	Better suited for integrated targeted biosensing because hollow lumens can deliver ISF directly to electrochemical, optical, colorimetric, fluorescence-based, or multiplexed sensors. Broad multi-omic analysis is more limited unless sufficient clean ISF is recovered into a separate reservoir.
Wearable Potential	Lower, as sampling generally involves multiple sequential steps.	High, as MN insertion, ISF collection, and readout can be integrated into one device.
Main Limitations	Multi-step workflow, vacuum seal integrity.	Fabrication complexity, lumen clogging, vacuum seal integrity.
Current Human Validation Status	More advanced human validation; ISF collection and downstream biomarker analyses have been reported in human studies.	Mostly animal validation; human ISF collection data remain limited.

## Data Availability

The original contributions presented in this study are included in the article. Further inquiries can be directed to the corresponding author.

## Abbreviations

The following abbreviations are used in this manuscript:

Ag/AgCl	Silver/silver chloride
CIM	Continuous immunoassay-based monitoring
CLIP	Continuous liquid interface production
CNN	Convolutional neural network
COP	Colloid osmotic pressure
COVID-19	Coronavirus disease 2019
CRP	C-reactive protein
DDAVP	Desmopressin acetate
DPV	Differential pulse voltammetry
DRIE	Deep reactive-ion etch
ECM	Extracellular matrix
ELISA	Enzyme-linked immunosorbent assay
Eu-MOF	Europium metal–organic framework
FEA	Finite element analysis
GAG	Glycosaminoglycan
GelMA	Gelatin methacrylate
GOx	Glucose oxidase
H&E	Hematoxylin and eosin
HMN	Hollow microneedle
HMNsAP	Hollow microneedle array patch
HRP	Horseradish peroxidase
IgG	Immunoglobulin G
IL-1β	Interleukin-1 beta
IL-6	Interleukin-6
ISF	Interstitial fluid
LC-MS	Liquid chromatography mass spectrometry
LC-MS/MS	Liquid chromatography tandem mass spectrometry
LFIA	Lateral flow immunochromatographic assay
LIFR	Laser-induced fluid recruitment
MAP	Microneedle array patch
MEMS	Micro-electro-mechanical systems
MN	Microneedle
MOF	Metal–organic framework
MPO	Myeloperoxidase
MS-RTIM	Microneedle sensor for real-time inflammation monitoring
nAbs	Neutralizing antibodies
PDMS	Polydimethylsiloxane
PMA	Polymethylacrylate
POP	Puncture-out-and-press
PVA	Polyvinyl alcohol
RGB	Red, green, blue
SARS-CoV-2	Severe acute respiratory syndrome coronavirus 2
SC	Stratum corneum
SEM	Scanning electron microscopy
SIADH	Syndrome of inappropriate antidiuretic hormone secretion
SPE	Screen-printed electrode
SU-8	Epoxy-based negative photoresist (trade name)
SUS	System usability scale
TMB	3,3′,5,5′-tetramethylbenzidine
TNF-α	Tumor necrosis factor alpha
VIES	Vacuum-integrated ISF extraction and sensing system
VT	Vacuum tube
WMNC	Wearable microneedle array-based colorimetric
